# Müller muscle–conjunctival resection in modern ptosis surgery: patient selection, technical refinements, and expanding indications

**DOI:** 10.3389/fsurg.2026.1950682

**Published:** 2026-09-24

**Authors:** Emirhan Ozkul, Mehmet Serhat Mangan

**Affiliations:** 1 Department of Ophthalmology, University of Health Sciences, Sancaktepe Prof. Dr. Ilhan Varank Education and Research Hospital, Istanbul, Türkiye; 2 Department of Ophthalmology, Division of Ocular Oncology and Oculoplastic Surgery, Istanbul Faculty of Medicine, Istanbul University, Istanbul, Türkiye

**Keywords:** blepharoptosis, external levator advancement, MMCR, müller muscle-conjunctival resection, phenylephrine test, posterior ptosis surgery, tarsectomy

## Abstract

Müller muscle-conjunctival resection (MMCR) has traditionally been regarded as a posterior approach for mild-to-moderate blepharoptosis in patients with good levator function and a positive response to phenylephrine. Its contemporary role, however, is considerably broader. Accumulating evidence supports the use of MMCR in selected patients with limited or absent phenylephrine responsiveness, more severe ptosis, postoperative eyelid asymmetry, and carefully chosen congenital, neurogenic, or neuromuscular forms of ptosis. In parallel, modifications that incorporate tarsectomy, simplified suturing, conjunctiva-sparing approaches, perioperative bandage contact lenses, and targeted hemostatic maneuvers have sought to improve predictability, comfort, and safety. Important uncertainties nevertheless remain. The mechanism by which MMCR elevates the eyelid is not fully explained by smooth-muscle excision alone; the predictive value of phenylephrine testing is inconsistent; and no single resection algorithm has proved universally reliable. Moreover, surgical success is still reported using heterogeneous definitions that may underrepresent eyelid contour, bilateral symmetry, ocular-surface health, and patient-reported benefit. This review examines the anatomical basis, patient-selection principles, operative algorithms, technical refinements, comparative outcomes, perioperative management, complications, and expanding indications of MMCR. Particular attention is given to the clinical settings in which posterior ptosis surgery may offer practical advantages over external levator advancement and to the limits of the available evidence. Modern MMCR is best understood not as a single formula-driven operation, but as an adaptable posterior lamellar platform whose results depend on individualized planning, meticulous execution, and assessment extending beyond eyelid height alone.

## 1 Introduction

Blepharoptosis is not simply a reduction in upper-eyelid height. Depending on its severity and cause, it may restrict the superior visual field, alter head posture, disturb ocular-surface protection, and create conspicuous asymmetry. Surgical correction therefore aims to restore a functional palpebral aperture while preserving a natural contour, an appropriate eyelid crease, bilateral symmetry, and safe closure. These goals are closely related but not interchangeable: a technically acceptable marginal reflex distance may coexist with an unsatisfactory contour, visible asymmetry, or postoperative surface symptoms (1).

For patients with preserved levator function, the two most widely used strategies are external levator advancement (ELA) and posterior lamellar ptosis repair. Müller muscle-conjunctival resection (MMCR), introduced by Putterman and Urist as a modification of earlier posterior procedures, gained popularity because it avoids a cutaneous incision, preserves the anterior lamella, and can provide a smooth eyelid contour with relatively limited dissection (2). Although the operation is named for the tissue that is resected, anatomical studies suggest that its effect cannot be attributed to removal of Müller smooth muscle alone. Posterior lamellar shortening, advancement or folding of the levator aponeurosis, and postoperative fibrosis probably contribute in varying degrees (3). This incomplete mechanistic explanation is clinically relevant because it weakens the assumption that pharmacological responsiveness of Müller muscle must determine the success of the operation.

The conventional candidate for MMCR has mild-to-moderate involutional ptosis, good levator excursion, and sufficient elevation after topical phenylephrine. Within this framework, several formulae have linked the observed pharmacological response and the amount of desired elevation to a predetermined length of conjunctiva and Müller muscle, with optional tarsectomy when additional elevation is required. Reported success rates are generally high, but the literature remains heterogeneous in patient selection, resection length, follow-up, and the definition of success. A recent comprehensive review, based on literature published through August 2023, reported success rates between 72% and 95% and an average marginal reflex distance 1 (MRD1) increase of approximately 2.1 mm (4). These summary values are clinically useful, yet they obscure meaningful variation between techniques and populations.

The role of the phenylephrine test has become a central point of debate. A positive response remains helpful for demonstrating achievable eyelid height, revealing contralateral Hering dependence, and discussing postoperative symmetry with the patient. It is less certain, however, whether the test is a prerequisite for MMCR or a sufficiently accurate dosing tool. In a multicenter cohort of 270 eyelids, preoperative phenylephrine testing did not significantly influence postoperative MRD1 change or reoperation rate; among tested eyelids, the postoperative position was predicted within 1 mm in only 60.2% (5). These findings do not make the test irrelevant, but they support a shift from a binary positive-vs.-negative rule toward interpretation alongside levator function, baseline asymmetry, ptosis severity, ocular motility, and surface risk.

The traditional boundary between anterior and posterior repair has also become less rigid. A systematic review and meta-analysis found broadly comparable eyelid elevation after MMCR and ELA, with MMCR associated with lower rates of overcorrection and reoperation in the included comparative studies (6). Retrospective data further suggest that MMCR can be effective in selected eyes with severe involutional ptosis when levator function is preserved (7). These findings should not be interpreted as evidence that MMCR is universally superior. ELA remains essential when direct inspection or adjustment of the levator aponeurosis is required, when major crease modification is planned, or when posterior lamellar surgery is undesirable. The more useful question is which approach best addresses the anatomy and priorities of an individual eyelid.

An expanding body of work has also positioned MMCR as a secondary or complementary operation. It has been used to correct residual unilateral ptosis following bilateral ELA and to treat contralateral ptosis that becomes clinically important after unilateral ELA (8, 9). In these settings, a posterior approach may restore symmetry without reopening a previously dissected anterior plane. The same principle has encouraged carefully selected applications in combined strabismus and ptosis surgery, corneal graft recipients, pediatric patients, and patients with neuromuscular ptosis.

Recent reports illustrate both the possibilities and the limits of this broader use. MMCR combined with tarsectomy has achieved useful elevation in selected patients with chronic progressive external ophthalmoplegia and partial phenylephrine responsiveness (10). Technical refinements have addressed perioperative comfort and uncommon but potentially serious bleeding, including a short traction-suture maneuver intended to provide temporary tamponade after resection (11). At the same time, a reported full-thickness eyelid defect following an otherwise routine MMCR is a reminder that a small-incision operation is not a risk-free operation and that clamp placement, tissue capture, suturing, and postoperative examination remain consequential (12).

The purpose of this review is therefore not to restate MMCR as an established treatment for phenylephrine-responsive involutional ptosis. Instead, it examines how contemporary evidence has changed patient selection, surgical planning, technique, perioperative care, and the interpretation of outcomes. Particular emphasis is placed on areas in which practice remains variable: the role of the phenylephrine test, the choice of resection algorithm, indications for tarsectomy, management of asymmetry and revision cases, expanding neurogenic and pediatric indications, ocular-surface safety, and prevention of complications. By integrating these issues, we aim to define where MMCR is predictable, where it is adaptable, and where the evidence still calls for restraint.

## 2 Literature search approach

This narrative review was informed by a targeted search of PubMed and reference chaining from key primary studies and recent reviews. Searches were updated through 26 July 2026 using combinations of the terms “Müller muscle-conjunctival resection,” “MMCR,” “conjunctivomullerectomy,” “blepharoptosis,” “phenylephrine,” “tarsectomy,” “external levator advancement,” “revision ptosis,” “ocular surface,” “complications,” “pediatric ptosis,” and “neuromuscular ptosis.” Peer-reviewed clinical studies, comparative studies, systematic reviews, anatomical investigations, and technically informative case series or reports were considered. Particular attention was given to evidence published after August 2023, which was the final literature-search date reported in the comprehensive MMCR review by Fatani et al. (4). Because the purpose was a clinically oriented state-of-the-art synthesis rather than a systematic review, study selection was guided by relevance to operative decision-making, technical evolution, outcome assessment, and unresolved controversy.

## 3 Anatomical and physiological basis of MMCR

### 3.1 The upper-eyelid elevator complex

The upper eyelid is elevated by a linked rather than a series arrangement of tissues. The levator palpebrae superioris provides the principal voluntary excursion. As the muscle approaches the orbital aperture, its aponeurosis spreads into an anterior layer that traverses the orbital septum and inserts into the pretarsal orbicularis and skin, and deeper fibers that are closely related to the superior tarsal border. Müller muscle arises from the undersurface of the levator complex, courses inferiorly immediately anterior to the conjunctiva, and inserts near the superior border of the tarsus. Its smooth-muscle fibers receive sympathetic innervation and provide a modest component of tonic eyelid elevation. The conjunctiva is firmly adherent to Müller muscle, whereas the plane between Müller muscle and the levator aponeurosis is comparatively loose. Microanatomical studies therefore support viewing the levator aponeurosis, Müller muscle, conjunctiva, and tarsus as a mechanically coupled elevator complex whose layers can move relative to one another (3, 13).

This anatomy helps explain why a purely pharmacological account of MMCR is insufficient. Sympathetic denervation, as in Horner syndrome, typically lowers the upper eyelid by only a few millimeters, whereas MMCR may produce a comparable or greater correction even when the preoperative phenylephrine response is incomplete. Conversely, a strong response to phenylephrine confirms recruitable elevation but does not specify which structural component will determine the postoperative position. Phenylephrine-induced elevation reflects the behavior of the intact eyelid system—including levator drive, posterior lamellar tension, orbital anatomy, and contralateral Hering dependence—rather than an isolated measurement of Müller muscle strength.

### 3.2 What does the operation actually resect?

In the standard closed technique, conjunctiva and Müller muscle are elevated from the posterior surface of the eyelid, incorporated within a clamp, sutured, and excised above the superior tarsal border. The specimen is consequently heterogeneous. It may contain conjunctiva, smooth muscle, vascular and fibrous tissue, and variable fragments of levator aponeurosis; when tarsectomy is added, a measured segment of superior tarsus is also removed. The proportion of each component depends on the dissection plane, tissue mobility, clamp position, and individual anatomy. Cadaveric simulation has shown that the levator aponeurosis can remain intact after MMCR, supporting the view that extensive levator excision is not required for elevation (3).

The variable composition of excised specimens is more than a pathological curiosity. It challenges the assumption that the millimeters marked on the conjunctival surface represent an equivalent dose of Müller muscle. In the histological analysis by Zauberman and colleagues, smooth muscle accounted for an average of only 21% of the excised tissue, and neither the amount nor the percentage of muscle correlated with the change in MRD1. The intraoperatively measured amount of composite tissue, rather than the quantity of smooth muscle within it, showed the more meaningful relationship with elevation (14). Resection length should therefore be understood as a practical surrogate for the amount of posterior lamella captured and shortened, not as a direct measurement of smooth-muscle removal.

### 3.3 A composite mechanism of eyelid elevation

Several mechanisms have been proposed, and none alone accounts for the full range of clinical observations. The original explanation emphasized resection and shortening of Müller muscle. A later interpretation described MMCR as an internal levator advancement: the resection and suture line displace the levator–Müller complex inferiorly and increase its effective attachment to the tarsus (15). This concept is attractive because most successful candidates have preserved levator function and because MMCR can correct aponeurotic ptosis without opening the anterior plane. It does not, however, require that a substantial segment of levator aponeurosis be present in the specimen.

A complementary explanation is mechanical shortening of the posterior lamella. Removing a composite strip of conjunctiva and Müller muscle reduces the distance between the superior fornix and tarsal plate. Closure then brings the proximal posterior lamella to the superior tarsal border, translating the border upward and folding or plicating the adjacent levator complex. The clamp and the transverse suture distribute this effect across the eyelid, which may help create the characteristically smooth contour. In this model, the operation changes the resting geometry and force transmission of the elevator complex; it does not simply remove a weak muscle.

Evidence from related posterior procedures reinforces this interpretation. Histological examination of eyelids corrected by the Fasanella–Servat operation showed that successful elevation could occur despite minimal or absent Müller muscle in the specimen, whereas tarsectomy and reattachment of the levator-related tissues were consistent findings (16). Similarly, the absence of a relationship between the percentage of excised smooth muscle and postoperative elevation after MMCR argues against a dose–response model based on Müller muscle alone (14). These observations do not mean that Müller muscle is irrelevant. Rather, it is both a contractile structure and an accessible posterior lamellar interface through which the surgeon can alter the position and tension of the levator–tarsal unit.

Postoperative wound healing probably adds a time-dependent component. The suture line creates adhesion between the shortened posterior lamella and the superior tarsal region, and subsequent collagen remodeling may stabilize the new relationship. This helps explain why the early eyelid position can change as edema resolves and why the definitive result cannot be inferred from the resected specimen alone. The relative contributions of immediate plication, levator coupling, and later cicatricial fixation are likely to differ among patients and between closed, open-sky, conjunctiva-sparing, and tarsectomy-assisted variants.

These anatomical relationships and the principal structural changes proposed to occur after MMCR are summarized schematically in Figure 1.

**Figure 1:**
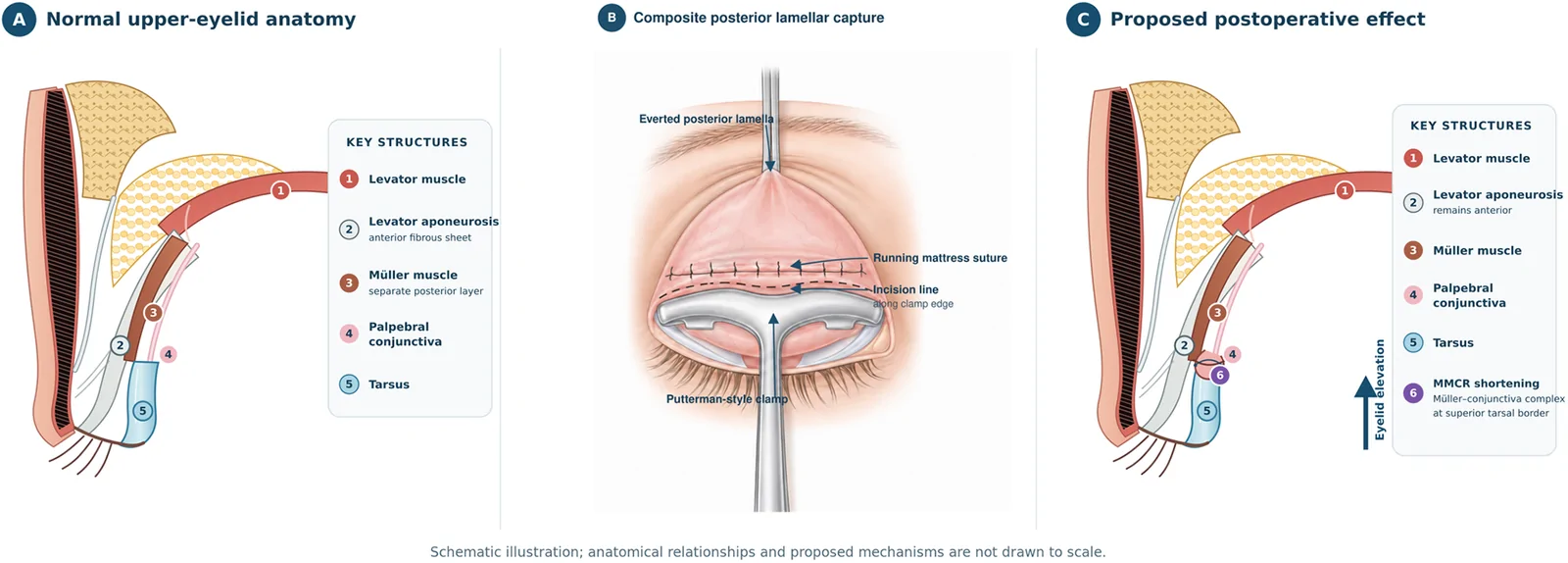
Anatomical, operative, and mechanistic overview of Müller muscle–conjunctival resection (MMCR). **(A)** Layered sagittal anatomy of the upper eyelid. The levator muscle belly continues anteriorly as the levator aponeurosis; Müller muscle is depicted as a distinct posterior layer arising from the undersurface of the distal levator and terminating at the superior tarsal border, with palpebral conjunctiva posteriorly. **(B)** Everted posterior lamella secured with a Putterman-style clamp; the running mattress suture is placed on the retained side, and the incision is made between the suture and the clamp while following the clamp edge. **(C)** Proposed Müller–conjunctival shortening at the superior tarsal border, with the levator aponeurosis remaining anterior. Anatomical relationships and proposed mechanisms are based on references 2, 3, and 14–16. The illustration is schematic and is not drawn to scale.

### 3.4 Histopathology and clinical response

Direct clinicopathological evidence remains limited but is beginning to refine this mechanistic model. In a 2025 prospective cohort of 47 patients, resected conjunctiva and Müller muscle were evaluated with hematoxylin–eosin and Masson trichrome staining. Greater Müller muscle fibrosis showed a modest positive association with ptosis correction, whereas muscle hypertrophy was not associated with the amount of correction or intereyelid difference (17). One proposed explanation is that a less elastic, more fibrotic posterior lamella may resist postoperative elongation and preserve the surgical shortening effect.

This finding is hypothesis-generating rather than a ready-to-use predictive test. The study was single-center, included a relatively small and etiologically mixed cohort, and assessed tissue only after the operative plan had already been executed. Fibrosis also correlated with the magnitude of correction but did not consistently separate success from failure across etiological subgroups. Routine pathology cannot therefore determine resection length preoperatively. Its more immediate value is conceptual: tissue quality, not only tissue quantity, may influence how posterior lamellar shortening is transmitted and maintained.

Taken together, the available anatomical, histological, and clinical data favor a composite model of MMCR. Eyelid elevation results from an interaction among posterior lamellar shortening, superior translation of the tarsal border, plication or advancement of the levator complex, and postoperative fibrotic stabilization. The patient's levator function, tissue compliance, baseline MRD1, Hering dependence, and ocular-surface status determine how that mechanical change is expressed. This model supports individualized planning and explains why both rigid phenylephrine-based rules and fixed millimeter-for-millimeter resection formulae perform imperfectly. It also provides the anatomical basis for the central clinical question addressed in the next section: whether phenylephrine responsiveness should remain a requirement for posterior ptosis surgery or one informative feature among several.

## 4 Patient selection and the changing role of the phenylephrine test

### 4.1 Beyond the classic ideal candidate

The classic MMCR candidate has mild-to-moderate ptosis, good levator function, a stable ocular examination, and sufficient eyelid elevation after topical phenylephrine. This profile remains useful because it identifies an eyelid in which a modest posterior lamellar intervention is likely to produce adequate elevation without direct anterior levator dissection. Historically, the phenylephrine response was also incorporated into formulae that selected a fixed conjunctiva–Müller muscle resection and added tarsectomy when further elevation was required (18). The apparent precision of these algorithms, however, should not obscure the variability of the tissues captured by the clamp or the patient-specific mechanical response described in the preceding section.

Contemporary selection is better organized around the eyelid phenotype than around a single test result. Baseline MRD1 should be measured with the brow relaxed and the head in a reproducible primary position. Levator excursion, eyelid crease height and quality, margin contour, tarsal stability, lagophthalmos, Bell phenomenon, orbicularis strength, corneal sensation, tear-film status, and the position of the fellow eyelid all influence the operative choice. Old photographs can clarify whether asymmetry is longstanding, whereas repeated measurements help identify fatigue or fluctuation that may indicate a neuromuscular disorder rather than stable aponeurotic ptosis. Visual-field limitation, brow recruitment, dermatochalasis, and the patient's priorities should also be recorded because normalization of MRD1 alone may not address the functional or aesthetic concern.

MMCR is particularly attractive when levator function is preserved, the desired change is predominantly in eyelid height, and avoiding or not reopening the anterior lamella is advantageous. This includes selected revision and symmetry procedures after ELA (8, 9). ELA may be preferable when the levator aponeurosis requires direct inspection, extensive contour adjustment is anticipated, the crease must be reconstructed, or concurrent skin and fat management is a central part of the operation. Poor levator function, marked ocular-surface vulnerability, weak protective mechanisms, significant conjunctival cicatrization, a shortened superior fornix, or unstable neurogenic or myogenic ptosis should prompt caution. These are not all absolute contraindications, but each reduces the value of a formula-driven posterior procedure and raises the importance of individualized risk assessment.

### 4.2 Performing and reading the test

A useful phenylephrine test begins with standardized examination rather than drop instillation. Both MRD1 values should be documented before testing, ideally with photographs obtained under consistent illumination and without frontalis recruitment. Phenylephrine 2.5% is then placed in the superior conjunctival fornix of the ptotic eye, and the eyelids are reassessed in primary gaze. Five- and ten-minute measurements capture the maximal response in most patients. In a detailed time-course study, 73% of tested eyes elevated; among responders, 50% reached maximal elevation by five minutes and 86% by ten minutes, although 14% did not reach their maximum until 30 min (19). A result recorded too early may therefore misclassify a delayed responder.

The test should be interpreted as a continuous response. The absolute post-phenylephrine MRD1, the change from baseline, the contour produced, and the relationship between the two eyelids are more informative than a binary positive or negative label. Pupillary dilation and conjunctival blanching do not reliably indicate that maximal eyelid elevation has occurred (19). Repeating the measurement after allowing additional time is reasonable when the clinical phenotype otherwise favors MMCR but the initial response is absent or equivocal. Variability in drop placement, baseline frontalis activity, photographic angle, examiner technique, and measurement to the nearest half millimeter can all materially change the apparent result.

### 4.3 What does phenylephrine actually predict?

Phenylephrine remains valuable as a simulation of direction: it demonstrates whether pharmacological recruitment of the posterior elevator complex can improve height and contour. It is less reliable as a millimeter-for-millimeter forecast. Ben Simon and colleagues found that phenylephrine testing underestimated the correction achieved with MMCR by approximately 40%, and final eyelid position was not explained by resection length alone (20). This discrepancy is consistent with a composite surgical mechanism that includes posterior lamellar shortening and cicatricial stabilization in addition to the reversible contraction produced by the drug.

More recent evidence further separates candidacy assessment from exact dosing. In the multicenter cohort described by Saffari and colleagues, postoperative MRD1 change, final MRD1, and reoperation rates were similar in eyelids operated with and without preoperative phenylephrine testing. Among tested eyelids, the postoperative position was predicted within 1 mm in 60.2% (5). The study does not establish that phenylephrine is unnecessary in every setting; it shows that performing the test does not by itself make the operation more predictable. A response can support the choice of MMCR and facilitate discussion with the patient, but it should not be treated as a calibrated measurement of the tissue that must be removed.

### 4.4 Does a negative test exclude MMCR?

A negative or limited phenylephrine response lowers confidence in the traditional algorithm but does not prove that posterior surgery will fail. In an early series of phenylephrine test-negative patients treated with open-sky MMCR, mean MRD1 increased by 3.3 mm and no patient required intraoperative augmentation (21). Other reports have similarly documented useful correction in selected test-negative eyelids. These studies are generally small, retrospective, and technique-specific; they support expansion of candidacy, not indiscriminate use.

The practical question after a negative test is whether the remaining anatomy still supports a posterior repair. Preserved levator function, a stable aponeurotic phenotype, acceptable ocular-surface reserve, and a modest correction target favor consideration of MMCR despite limited pharmacological elevation. Conversely, poor levator excursion, a very low baseline MRD1, severe congenital dysgenesis, significant exposure risk, or the need for extensive crease and contour reconstruction favors an alternative or combined approach. The decision should also account for the surgeon's technique: evidence derived from an open-sky dissection or tarsectomy-assisted operation cannot automatically be transferred to every closed-clamp resection.

### 4.5 Bilateral planning and Hering dependence

The most durable role of phenylephrine may be assessment of bilateral relationships. In a multicenter prospective cohort of 229 eyelids (22), MMCR achieved the prespecified MRD1 criterion in 72.1% of eyelids and intereyelid symmetry in 75.7% of patients. Baseline MRD1 before phenylephrine was the strongest predictor of height success, whereas symmetry after phenylephrine increased the odds of postoperative symmetry. A large drug-induced rise and a longer resection did not fully compensate for a very low baseline MRD1. Thus, the post-phenylephrine relationship between the eyelids may be more clinically informative than the response amplitude of the ptotic eyelid considered alone.

The fellow eyelid must nevertheless be interpreted cautiously. In a prospective series of unilateral MMCR (23), a clinically relevant late Hering response occurred in 8% of patients. A contralateral descent provoked by preoperative phenylephrine did not reliably predict the late postoperative response; immediate and one-week postoperative changes were more informative. These data argue against automatically operating on a clinically normal fellow eyelid solely because it descends during testing. They also support explicit preoperative counseling that unilateral repair can reveal or accentuate contralateral ptosis and that staged management may occasionally be preferable.

Phenylephrine should therefore be retained as one component of a structured examination, not as a mandatory gatekeeper. It is useful for demonstrating a possible contour, assessing bilateral symmetry, revealing Hering dependence, and framing expectations. Its limitations are equally important: response timing varies, measurement error is common, test-negative eyelids may still respond to surgery, and the result does not dictate an exact resection length. Modern patient selection integrates the test with baseline MRD1, levator function, etiology, tissue quality, ocular-surface safety, fellow-eyelid behavior, and the specific goals of surgery.

## 5 Resection algorithms, tarsectomy, and surgical decision-making

### 5.1 From historical nomograms to surgical families

Resection algorithms were developed to convert a qualitative examination into a reproducible operative plan. The classic schemes linked the amount of ptosis, the phenylephrine response, and the length of posterior lamellar tissue placed within the clamp. Dresner proposed a semilinear formula with further adjustment when the phenylephrine response was incomplete (24). Perry and colleagues subsequently used a 9-mm conjunctiva–Müller muscle resection as the base operation and added a measured tarsectomy corresponding to the residual undercorrection after phenylephrine (18). These nomograms were clinically influential because they replaced an impressionistic decision with an explicit starting point.

The apparent numerical precision should not be mistaken for equivalence among techniques. Published procedures differ in the landmark from which tissue is measured, the amount tented into the clamp, whether the levator aponeurosis is incorporated, and whether tarsus is resected. A nominal 8-mm resection therefore does not necessarily represent the same anatomical intervention in every series. In practice, contemporary posterior repair can be organized into three broad families: a fixed tarsus-sparing resection, a variable tarsus-sparing resection adjusted to the desired elevation, and a conjunctiva–Müller muscle resection augmented by tarsectomy. Standardization within each family improves consistency, but direct transfer of a millimeter rule between surgical techniques remains problematic.

### 5.2 Is resection length a true dose?

A strict dose–response model predicts that each additional millimeter of resection should produce a stable increment in eyelid elevation. Clinical data do not support that degree of precision. Earlier studies found that final eyelid position was only weakly related, or unrelated, to the amount of conjunctiva and Müller muscle removed (14, 20). In a large single-center analysis, Rootman and colleagues likewise found that tissue resection length was not a dominant determinant of postoperative position once baseline anatomy and phenylephrine response were considered (25).

The same group later compared a standard 7-mm resection with a variable nomogram using approximately 4 mm of resection for each millimeter of desired elevation (26). The difference between the groups was only 0.1 mm in final MRD1 and 0.2 mm in MRD1 change, neither of which was statistically significant. This does not mean that resection length is irrelevant at all extremes. It means that small adjustments within the usual operative range may be overwhelmed by tissue compliance, levator coupling, wound contraction, measurement error, and the bilateral neural response.

A later comparison of 95 patients with asymmetric ptosis reached a similar conclusion (27). Variable bilateral resection, fixed bilateral 7-mm resection, and fixed resection with 1-mm tarsectomy on the lower side all improved symmetry, but the selected algorithm did not independently predict postoperative asymmetry. Preoperative asymmetry was the only significant predictor in multivariable analysis. Thus, resection length is best viewed as a coarse surgical control rather than a calibrated pharmacological dose. A surgeon-specific range can be reproducible, but its expected effect must be interpreted within the phenotype and the technique in which it was developed.

### 5.3 Tarsectomy as selective augmentation

Tarsectomy changes the operation more substantially than a small increase in conjunctiva–Müller muscle resection. Removing superior tarsus shortens a rigid component of the posterior lamella and can provide additional elevation when the phenylephrine result or a standard MMCR is unlikely to reach the target. It is therefore conceptually useful as an augmentation step, rather than as the automatic continuation of a linear resection scale. The Perry algorithm formalized this distinction by keeping the conjunctiva–Müller component constant and assigning the residual correction to tarsus (18).

Severe ptosis alone, however, does not mandate tarsectomy. In a series of severe involutional blepharoptosis, MMCR with or without tarsectomy produced substantial elevation; among eyelids treated without tarsectomy, mean MRD1 increased from −0.51 to 2.98 mm and 97.5% achieved more than 1.5 mm of elevation (28). These results support posterior repair in selected severe cases with preserved levator function, while also showing that baseline severity cannot by itself determine which tissue must be removed.

The most direct evidence for an incremental tarsal effect comes from a study of 60 patients undergoing 8-mm MMCR with either 1- or 2-mm tarsectomy (29). Mean MRD1 elevation was 1.66 mm after 1-mm tarsectomy and 2.72 mm after 2-mm tarsectomy; symmetry within 1 mm was achieved in 90.6% and 85.7%, respectively. The authors proposed an expected elevation of 2.1 mm as a practical threshold for choosing between the two tarsal lengths. The study supports a measurable augmentation effect, but its retrospective design, unilateral population, and single operative method limit conversion of these values into a universal formula.

The additional lift must be balanced against the loss of structural and ocular-surface reserve. Tarsal height should be measured rather than assumed, and a conservative central resection should preserve an adequate marginal segment. Clamp placement must also avoid unintended full-thickness eyelid capture, a rare but potentially serious complication illustrated in the authors’ clinical experience (12). Tarsectomy deserves particular caution in patients with short tarsi, conjunctival scarring, marginal instability, lagophthalmos, reduced corneal sensation, or limited tear-film reserve.

### 5.4 Extending posterior surgery to severe and atypical ptosis

Recent algorithms have deliberately extended posterior surgery beyond mild, phenylephrine-responsive involutional ptosis. In a prospective interventional series of 118 procedures in 81 adults with moderate-to-severe ptosis and at least fair levator function (30), the planned resection combined 3–12 mm of conjunctiva and Müller muscle with tarsectomy for residual ptosis while preserving at least 4 mm of upper tarsus. Mean MRD1 increased from 0.2 to 4.1 mm, and intereyelid symmetry within 1 mm was reported in 95% of procedures. These results are clinically important, but the algorithm used 10% phenylephrine and a specific extended posterior technique; both the efficacy and safety boundaries require external validation before routine adoption.

A separate series treated MMCR as a first operative step across varying etiologies and severity. Among 34 patients and 56 eyelids, mean MRD1 increased from 1.3 to 3.2 mm, and only two patients had postoperative asymmetry greater than 1 mm (31). The appeal of this strategy is its staged logic: a relatively limited posterior operation may achieve the desired height while preserving the anterior lamella for later adjustment if necessary. Its evidence remains level IV, and patients who require major crease reconstruction, broad contour modification, or direct aponeurotic inspection may still be better served by an anterior or combined procedure.

Etiology becomes especially important when the posterior approach is extended. The authors’ experience with MMCR and tarsectomy in chronic progressive external ophthalmoplegia illustrates that useful elevation can be obtained in a carefully selected myogenic eyelid (10). Such cases should not be interpreted as evidence for formula-driven tarsectomy in all myogenic ptosis. The target height must remain conservative, and preoperative Bell phenomenon, orbicularis function, lagophthalmos, corneal sensation, fatigue, and the expected course of the underlying disease may be more important than the nominal resection length.

### 5.5 A practical hierarchy for resection planning

A useful contemporary algorithm begins with the intended postoperative relationship between the eyelids, not with the clamp measurement. Baseline MRD1, desired elevation, levator function, contour, crease, tarsal height, ocular-surface reserve, and the fellow eyelid response define the safe target. The surgeon then selects the operative family. A fixed tarsus-sparing resection is reasonable for a typical eyelid requiring modest elevation; a variable resection may be used when it reflects a surgeon's validated experience; and tarsectomy is reserved for selected eyelids requiring additional shortening with sufficient tarsal and surface reserve.

Standardized phenylephrine-guided planning can still be effective within a well-defined population. In a 2026 series of 59 patients undergoing bilateral MMCR for mild-to-moderate ptosis with good levator function and positive phenylephrine responses (32), mean MRD1 improved from approximately 1.3 mm in each eye to approximately 2.6–2.7 mm at three months. Excellent symmetry was reported in 74.6%, satisfactory symmetry in 20.3%, and poor symmetry in 5.0%. The result supports disciplined bilateral planning, but its narrow eligibility criteria and short follow-up should not be used to reinstate phenylephrine as a universal gatekeeper or the nomogram as an exact dose.

The operative amount should ultimately be understood as an informed starting estimate. When the predicted lift approaches the limit of posterior surgery, a conservative staged plan may be preferable to maximal one-stage resection, particularly in an ocular-surface vulnerable or myogenic eyelid. Conversely, a modest undercorrection can usually be reassessed after edema and wound remodeling have stabilized, whereas overcorrection with exposure may demand urgent intervention. This asymmetry of risk is central to responsible dose selection.

No current formula has been validated across all ethnicities, etiologies, degrees of ptosis, levator functions, and technical variants. The most defensible approach is therefore structured but flexible: define the phenotype, use a familiar and documented resection family, add tarsus selectively, and audit personal outcomes against a standardized photographic endpoint. Future prospective studies should compare algorithms using identical measurement conventions and should report not only MRD1, but also contour, symmetry, ocular-surface outcomes, reoperation, and patient-reported benefit.

## 6 Operative technique and contemporary technical modifications

### 6.1 Core surgical sequence and reproducibility

The apparent simplicity of MMCR can obscure the importance of a disciplined operative sequence. A conventional closed-clamp procedure begins with controlled eyelid eversion and clear identification of the superior tarsal border. The planned resection is marked on the palpebral surface, the conjunctiva–Müller complex is elevated symmetrically, and the intended tissue is secured before excision. Closure then brings the cut posterior lamellar edges into stable apposition. These steps are familiar, but their execution determines whether the operation behaves as a reproducible posterior shortening procedure or as an uncontrolled composite resection.

Three technical variables deserve particular attention. First, the measurement must refer to a consistent landmark; the distance marked from the superior tarsal border is not interchangeable with the amount of tissue ultimately contained within the clamp. Second, the central and lateral tissue bites should be balanced to avoid an oblique resection and an unintended contour peak. Third, the surgeon should inspect both surfaces of the everted eyelid before cutting. The clamp must contain the intended posterior tissue without skin, orbicularis, lashes, or the full thickness of the eyelid margin. Surgical microanatomy studies show that the excised specimen may include variable amounts of levator-related tissue as well as conjunctiva and Müller muscle; precise tissue control is therefore more meaningful than the nominal millimeter value alone (3).

The sequence should also protect the globe and ocular surface. Instruments and sutures must remain under direct control while the eyelid is everted, and the cornea should not be exposed to a sharp cut edge or an unburied knot. Hemostasis should be confirmed before the eyelid is returned to its anatomical position. At the end of surgery, eyelid height cannot be judged as reliably as during an anterior levator adjustment because edema, local anesthetic, and the fixed posterior wound alter the immediate position. Reproducibility therefore depends on preoperative planning, consistent clamp placement, and postoperative audit rather than repeated intraoperative height adjustment.

### 6.2 Closed-clamp, open-sky, and conjunctiva-sparing approaches

The classic closed-clamp operation remains the reference technique because it is rapid, requires limited dissection, and can produce a smooth transverse shortening when the tissue is captured evenly (2). Its limitation is that the exact contribution of Müller muscle, levator aponeurosis, and adjacent connective tissue is not directly visualized. That limitation is usually acceptable in a typical aponeurotic eyelid with good levator function, but it becomes more consequential when anatomy is scarred, the phenylephrine response is limited, or a previous operation has altered tissue planes.

Open-sky posterior surgery replaces blind tissue capture with direct dissection. The conjunctiva and Müller muscle can be separated, the levator complex identified, and the desired tissue advanced or shortened under visualization. This flexibility may help in selected phenylephrine-negative or revision cases. In the small open-sky series discussed earlier, test-negative eyelids achieved a mean MRD1 increase of 3.3 mm (21). The result supports anatomical rather than pharmacological selection in carefully chosen patients, but does not prove superiority over the closed technique. Open dissection takes longer, introduces additional tissue handling, and depends more heavily on the surgeon's ability to identify altered posterior lamellar planes.

Conjunctiva-sparing posterior repair takes the concept further by separating and preserving conjunctiva while shortening the Müller–levator interface. The rationale is attractive: retention of healthy conjunctiva and avoidance of a conjunctival suture line may reduce foreign-body exposure and preserve ocular-surface tissue. In a retrospective series of 100 patients and 171 eyelids undergoing a sutureless conjunctiva-sparing Müllerectomy, mean MRD1 improvement at six months was 2.85 mm, symmetry within 1 mm was observed in 91% of cases, and mean operative time was shorter than for traditional MMCR (33). These results establish feasibility, not universal advantage. The study was single-center and nonrandomized, and preservation of conjunctiva should not be assumed to eliminate dry-eye symptoms or exposure risk. The choice among closed, open-sky, and conjunctiva-sparing techniques should therefore reflect the anatomy, surface reserve, required degree of visualization, and the surgeon's validated experience.

### 6.3 Sutures, sealants, and sutureless closure

Traditional MMCR uses a running or mattress absorbable suture to approximate the posterior lamellar edges and distribute shortening across the eyelid. The suture is not merely a closure device: bite depth, transverse tension, and knot position can influence contour and early corneal irritation. Technical modifications have therefore attempted to reduce the amount of exposed material without losing wound stability. A single-suture MMCR modification produced postoperative elevation, safety, satisfaction, and revision rates comparable with those of traditional MMCR in a two-center retrospective analysis (34). The practical attraction is a shorter, simpler closure; the evidence does not show that one suture pattern is intrinsically more accurate in every eyelid.

Sutureless Müllerectomy removes the foreign-body component altogether, usually relying on controlled tissue apposition and thermal or mechanical wound sealing. An early series reported 34 sutureless procedures in 19 patients and described the method as rapid and effective (35). A later comparison of 56 eyelids found a larger mean MRD1 change after sutureless than conventional MMCR (2.74 vs. 2.02 mm), while changes in vertical lid height and postoperative symmetry were similar (36). The groups were retrospective and had different follow-up durations; the numerical difference should therefore not be interpreted as proof that omission of sutures increases the surgical dose. These studies more reasonably suggest that a stable posterior wound can be achieved without a traditional running suture in selected hands.

Fibrin sealant represents an intermediate strategy: the resection is performed in the usual manner, but the cut edges are approximated with a biologic adhesive rather than transconjunctival suture. In a retrospective comparison of 367 eyelids, fibrin sealant produced eyelid position and symmetry equivalent to suture closure and was associated with fewer major postoperative complications (37). The approach may reduce suture-related discomfort and shorten closure time, but cost, availability, handling, and the theoretical risks of blood-derived products limit universal adoption. Across all closure methods, current evidence is dominated by retrospective studies and technique-specific series. A new closure should be judged by stable height and contour, wound integrity, ocular-surface comfort, reoperation, and total operative burden rather than by the absence of sutures alone.

### 6.4 Hemostasis, tissue control, and prevention of technical error

Bleeding is usually limited, but it can disrupt visualization, conceal an uneven wound edge, and occasionally recur after the early postoperative period. Prevention begins before excision: tissue should be elevated without tearing, the clamp should apply uniform compression, and the cut should remain immediately adjacent to the instrument. After clamp release, the wound must be examined rather than assumed to be dry. Targeted bipolar coagulation can control a focal vessel, but excessive thermal treatment may increase posterior lamellar scarring and surface inflammation.

The authors have described a simple traction-suture maneuver intended to add mechanical tamponade to this sequence. After resection, a 6–0 polypropylene suture was gently tensioned for approximately one minute before being secured over a bolster. In a retrospective series of 23 patients, mean MRD1 improvement was 2.2 mm and no intraoperative or postoperative hemorrhage was observed (11). The study is small and has no control group, so it cannot define the incidence reduction attributable to the maneuver. It nevertheless illustrates a useful technical principle: brief, evenly distributed compression may provide hemostasis without repeated cautery or prolonged operative time.

Avoidance of unintended tissue capture is equally important. A recently reported full-thickness eyelid defect became apparent after suture removal following a 9-mm closed-clamp MMCR and required revision (12). The rarity of this event should not diminish its instructional value. Before excision, the surgeon should confirm free mobility of the anterior lamella, inspect the lash line and skin surface, and verify that the clamp has not incorporated the eyelid margin. Tarsectomy requires an additional check of residual tarsal height and central alignment. When the tissue plane or clamp contents are uncertain, releasing and replacing the instrument is safer than completing an irreversible cut.

Technical safety also extends to postoperative instructions. Patients should be warned that increasing pain, sudden bleeding, reduced vision, marked swelling, or new diplopia is not an expected expression of routine edema. Early examination should assess the cornea, wound, eyelid contour, and downgaze as well as primary position. The combination of careful tissue capture, deliberate hemostasis, and an explicit escalation pathway is more protective than any single instrument or suture choice.

### 6.5 Postoperative ocular-surface protection and comfort

The posterior position of the wound makes ocular-surface comfort a technical outcome, not a secondary cosmetic concern. Early symptoms may arise from exposed suture, a rough conjunctival edge, transient tear-film instability, topical medication, or incomplete closure. The cornea should be examined at the end of surgery and during early follow-up, particularly in patients with dry eye, reduced corneal sensation, previous refractive or corneal surgery, incomplete blink, or combined blepharoplasty.

A prospective randomized study by Mangan and colleagues directly compared a bandage contact lens with eye patching after unilateral MMCR in 40 patients (38). At postoperative days 1 and 7, the contact-lens group had lower pain scores and anti-inflammatory medication use, less corneal fluorescein staining, and better tear break-up time; all between-group differences were statistically significant. The study provides clinically relevant evidence that covering the posterior wound–cornea interface can improve early comfort and surface findings while allowing binocular daily activity.

A bandage lens should still be individualized. Correct fit, intact epithelium, adequate follow-up, and clear infection precautions are essential, and routine use may be inappropriate in patients unable to manage a lens or return promptly for review. When an externalized nonabsorbable suture or bolster is used, the duration of lens protection and timing of removal should be coordinated with the planned suture removal. With absorbable, buried, sealant-assisted, or sutureless techniques, lubrication alone may be sufficient in a low-risk surface.

Contemporary technique should therefore be evaluated across two linked endpoints. The first is mechanical: height, contour, symmetry, and wound stability. The second is biological and experiential: epithelial integrity, tear-film behavior, pain, visual function, and return to daily activity. Innovations that shorten operating time but increase surface morbidity are not true improvements; conversely, a small technical modification that reliably reduces discomfort without compromising eyelid position may represent meaningful patient-centered progress.

## 7 Clinical outcomes, complications, and revision strategies

### 7.1 Defining success beyond eyelid height

Reported success after MMCR is highly sensitive to the endpoint chosen. Some series define success by a postoperative MRD1 within a specified numerical range; others require inter-eyelid asymmetry of 1 mm or less, absence of reoperation, or a combination of these criteria. A patient may therefore be classified as a success in one study and a failure in another despite an identical postoperative appearance. This heterogeneity helps explain the broad range of published success rates and limits direct comparison between algorithms, techniques, and surgical centers (4, 22).

MRD1 remains indispensable, but it is only one dimension of the result. A high eyelid with a temporal droop, a central peak, an altered crease, or impaired closure is not an optimal outcome. Conversely, an eyelid that falls just outside an arbitrary MRD1 interval may be functionally and aesthetically satisfactory if its contour is natural and it is well matched to the fellow side. The most meaningful assessment combines standardized primary-gaze photographs, MRD1, inter-eyelid difference, contour, crease height, lagophthalmos, corneal staining, visual-field or head-posture improvement when relevant, and the patient's perception of symmetry and function.

Timing is equally important. Early edema, reflex frontalis recruitment, and transient surface irritation can distort both height and patient-reported comfort. A useful audit therefore separates early safety from stable effectiveness: the first postoperative visits identify epithelial injury, bleeding, infection, wound problems, and marked overcorrection, whereas final height and contour should be judged only after the eyelid has settled. Studies that report a single early measurement risk overstating both success and failure. A proposed minimum framework for standardized MMCR outcome reporting is summarized in Table 1.

**Table 1:** Proposed minimum outcome-reporting framework for future MMCR studies.

Domain	Minimum recommended variables	Reporting considerations
Baseline phenotype	Etiology; unilateral/bilateral involvement; MRD1 in both eyes; intereyelid difference; levator function; phenylephrine protocol and response; previous eyelid surgery; ocular-surface risk	Report eligibility criteria and operative technique clearly
Eyelid position and symmetry	Postoperative MRD1 in both eyes; change in MRD1; intereyelid difference; undercorrection; overcorrection	Prespecify the target MRD1 range; report individual components as well as composite success
Contour and eyelid anatomy	Standardized bilateral photographs; predefined contour assessment; crease position; lagophthalmos; eyelid closure	Use primary gaze, frontalis relaxation, consistent illumination, and image scale
Ocular surface and safety	Corneal fluorescein staining; dry-eye symptoms; epithelial injury; hemorrhage; infection; symblepharon; other adverse events	Separate early postoperative safety from stable outcomes
Patient-reported benefit	Perceived symmetry; functional improvement; aesthetic satisfaction; discomfort and recovery	Use a validated patient-reported instrument when available
Revision and follow-up	Revision rate and indication; time to revision; duration of follow-up	Report stable effectiveness at ≥3 months and longer-term durability separately
Unit of analysis	Per-eyelid and per-patient results	Account for within-patient correlation in bilateral procedures

### 7.2 MMCR compared with ELA

The choice between MMCR and ELA should not be framed as a contest between universally competing operations. ELA provides direct access to the levator aponeurosis, permits intraoperative adjustment, and is adaptable when the crease, anterior lamella, or levator anatomy requires reconstruction. MMCR avoids an external incision, preserves the anterior lamella, and offers a relatively standardized operation whose final position is planned before tissue removal. The appropriate comparison is therefore phenotype-specific rather than procedure-wide.

The 2023 systematic review and meta-analysis found broadly comparable efficacy between MMCR and ELA, with MMCR showing advantages in overcorrection and reoperation outcomes in the pooled literature (6). In a separate series restricted to severe involutional ptosis with a good phenylephrine response, MMCR achieved high success and compared favorably with ELA despite the traditional assumption that a posterior operation should be reserved for mild disease (7). These findings support broader use, but they do not erase selection bias: surgeons commonly direct more complex eyelids, lower levator function, and altered anterior anatomy toward ELA.

A retrospective review of 1,519 ptosis operations reported revision in 6.8% of MMCR cases and 9.5% of ELA cases; the overall revision rate was 8.7%, and undercorrection was the most frequent reason for reoperation (39). The result is clinically useful but should not be interpreted as a randomized estimate of superiority, because baseline ptosis was greater in the ELA group. It does, however, challenge the assumption that the inability to adjust MMCR intraoperatively necessarily produces more revisions.

Operative efficiency and training are additional considerations. Cumulative sum (CUSUM) analysis is a sequential monitoring method that plots accumulated deviations from a predefined target and can identify changes in performance and the point at which operative performance stabilizes. In a CUSUM analysis of the first 30 unilateral ELA and 30 unilateral MMCR procedures performed by one surgeon (40), operating time stabilized after approximately 11 ELA and 12 MMCR cases. ELA nevertheless had longer operating time, more intraoperative complications, lower postoperative success, and more reoperations in that early experience. The nearly identical stabilization points caution against describing MMCR as a procedure that requires little training. Its steps are shorter, but consistent tissue capture, contour control, and patient selection still require deliberate supervision and outcome audit.

### 7.3 Undercorrection, overcorrection, asymmetry, and reoperation

Undercorrection is the most common unsatisfactory height outcome after MMCR. Potential causes include an inadequate biological response, insufficient posterior shortening, uneven tissue capture, unrecognized levator dysfunction, postoperative attenuation, or a contralateral Hering-related change that makes the operated eyelid appear relatively low. Before planning revision, the surgeon should allow stable healing, repeat measurements with the brow relaxed, compare current findings with standardized preoperative images, and determine whether the problem is true low height, contour abnormality, or bilateral imbalance.

Both repeat MMCR and ELA are defensible when the eyelid remains low after an initial MMCR. In a small comparative revision series, each strategy produced acceptable elevation and final position with low complication rates (41). Repeat MMCR is most attractive when the posterior lamella remains healthy, levator function is good, the contour is otherwise satisfactory, and a modest additional elevation is required. ELA is preferable when the crease or contour needs direct reconstruction, the initial response suggests inadequate levator coupling, substantial elevation is required, or another posterior resection would threaten the conjunctiva or tarsus.

A separate series of repeat MMCR in 12 eyelids found a mean additional MRD1 elevation of approximately 1 mm, supporting the feasibility of a second posterior procedure in selected patients (42). That average also defines its limitation: repeat MMCR should not be promised as a large-dose rescue operation. The residual tarsal height, previous resection length, surface status, and location of the first scar must be known before another clamp or resection is applied.

Overcorrection is less common but potentially more urgent because lagophthalmos and exposure can threaten the cornea. Mild early elevation may improve as edema resolves; lubrication, temporary moisture protection, and close review are appropriate when the epithelium is intact. Progressive pain, staining, epithelial defect, or visually significant height requires prompt intervention. Depending on timing and anatomy, options include wound release, removal or adjustment of an accessible suture, posterior lamellar relaxation, or levator recession. The threshold for intervention should be lower in a neurotrophic cornea, previous graft, poor Bell phenomenon, or pre-existing exposure.

Contour abnormalities and asymmetry require diagnosis rather than reflexive additional resection. A central peak may reflect a disproportionately tight central bite; temporal droop may represent inadequate lateral capture or unrecognized lateral aponeurotic attenuation. When the height is acceptable but contour is not, focal release or anterior contour adjustment may be more appropriate than repeating the original dose across the entire eyelid.

### 7.4 Ocular-surface and conjunctival morbidity

Removal of palpebral conjunctiva and manipulation of accessory lacrimal tissue create a plausible concern for dry eye, yet the clinical literature is mixed. Quantitative studies have generally not demonstrated a uniform, sustained deterioration in tear production or surface parameters after uncomplicated MMCR. In one prospective evaluation, standard dry-eye measures did not show clinically important worsening after surgery, although individual patients could still experience symptoms during early healing (43). The correct conclusion is not that MMCR is surface-neutral, but that average group data cannot replace individual risk assessment.

The conjunctiva also remodels after resection. Histological comparison of previously operated and unoperated eyelids identified postoperative conjunctival changes and reduced goblet-cell density in the small specimens available (44). The study was based on only a few repeat-surgery samples, so it cannot quantify clinical dry-eye risk. It nevertheless supports preserving healthy tissue when possible and avoiding repeated posterior surgery without a clear benefit.

The greatest caution applies to a scar-prone or chronically inflamed conjunctiva. Diplopia and symblepharon have been reported after MMCR in patients receiving multiple long-term topical glaucoma medications (45). Chronic preservative exposure, prior filtering surgery, cicatrizing disease, and fornix shortening should therefore be actively sought. In these patients, a posterior approach may sacrifice tissue needed for future glaucoma care or convert subclinical inflammation into symptomatic scarring. ELA, a conjunctiva-sparing modification, or observation may be safer.

Practical surface prevention begins before surgery: document symptoms, tear-film stability, staining, corneal sensitivity, blink, Bell phenomenon, lagophthalmos, topical medication burden, and previous ocular procedures. Treat modifiable disease first, limit simultaneous skin excision when closure reserve is uncertain, bury or eliminate exposed suture, and provide a lubrication plan matched to risk. Postoperative review should include fluorescein staining and symptom inquiry even when MRD1 is excellent.

### 7.5 Hemorrhage and rare but consequential complications

Delayed hemorrhage deserves specific counseling because its timing may be unexpected. In a five-year case-control review (46), 10 of 350 patients developed hemorrhage more than 24 h after MMCR, an incidence of 2.9%. Bleeding began a mean of 4.2 days after surgery, lasted 12–72 h, and resolved with conservative measures in all cases; no consistent medical or medication-related risk factor was identified. Patients should therefore receive clear instructions even when the first postoperative examination is reassuring.

Initial management depends on severity. The patient should be assessed for visual loss, proptosis, tense orbit, afferent pupillary defect, severe pain, corneal compromise, and ongoing wound bleeding. External pressure and observation may be sufficient for limited posterior wound hemorrhage, but suspected orbital compartment syndrome requires emergency decompression. Antithrombotic and supplement history should be reviewed without assuming that discontinuation is safe; perioperative changes require coordination with the prescribing clinician.

A 2026 case report from the authors' group described recurrent late bleeding on postoperative days 5 and 6 that was managed, after hematology consultation, with oral tranexamic acid (47). This observation is hypothesis-generating rather than a protocol: tranexamic acid has contraindications and thrombotic considerations, and evidence from one case cannot establish routine use. Its value is to emphasize multidisciplinary evaluation when bleeding is persistent or recurrent despite local measures.

Other uncommon complications include corneal abrasion or ulceration, infection, granuloma, retained or migrated suture, entropion, lash malposition, fornix scarring, diplopia, and unintended full-thickness eyelid injury. Their rarity makes incidence estimates imprecise, but many share preventable mechanisms: incorrect tissue capture, exposed material, excessive cautery, unrecognized surface disease, or delayed response to warning symptoms. A complication checklist is therefore more useful than describing MMCR as intrinsically minor surgery.

## 8 Expanding indications and integration in modern oculoplastic practice

### 8.1 Secondary and symmetry surgery after ELA

One of the most practical expansions of MMCR is its use as a complementary operation after ELA. Bilateral involutional ptosis may present asymmetrically, and correction of the more ptotic eyelid can reveal either residual ipsilateral ptosis or contralateral descent. Re-entering the anterior plane may disturb a satisfactory crease and contour; a posterior correction can add height while preserving the previous anterior reconstruction.

Mangan and colleagues reported effective MMCR for unilateral residual ptosis after bilateral ELA, with improved inter-eyelid symmetry in appropriately selected patients (8). The same group later used MMCR for contralateral ptosis that emerged after unilateral ELA (9). Together, these studies support viewing MMCR and ELA as complementary tools rather than mutually exclusive primary procedures. The posterior approach is particularly useful when levator function is good, the residual deficit is modest, the contour and crease are acceptable, and phenylephrine or clinical elevation suggests sufficient reserve.

Secondary posterior surgery should still be individualized. A distorted crease, marked temporal droop, levator dehiscence, or large residual deficit usually favors ELA. Bilateral measurements must be repeated after frontalis relaxation, and Hering dependence should be discussed before a seemingly unilateral touch-up. The goal is not simply to avoid the first surgical plane, but to choose the plane that directly addresses the remaining abnormality.

### 8.2 Severe, congenital, and pediatric ptosis

Severe ptosis is no longer an absolute boundary for MMCR when levator function and phenylephrine responsiveness are favorable. Studies incorporating longer posterior resections or selective tarsectomy have achieved useful elevation in severe involutional ptosis (7, 28). The key discriminator is not baseline MRD1 alone. An eyelid with severe aponeurotic ptosis but good excursion and a strong pharmacological response may be more suitable for posterior surgery than an eyelid with milder height loss but poor levator function and weak closure.

Congenital ptosis requires greater caution because levator dysgenesis, contour abnormality, lag in downgaze, amblyopia risk, and long-term growth all affect the operative choice. In a series of 38 eyelids in patients aged 2–25 years, MMCR with tarsectomy was reported as safe and effective for congenital ptosis with moderate-to-good levator function, with follow-up extending to four years and no reported complications (48). The findings support a selected subgroup, not replacement of levator resection or frontalis suspension in poor-function congenital ptosis.

A 2026 retrospective pediatric study further evaluated MMCR in patients younger than 18 years with mild-to-moderate ptosis, good levator function, and a positive 2.5% phenylephrine test (49). Functional or developmental indications included visual-axis obstruction, abnormal head posture, significant asymmetry, and psychosocial concern. This contemporary framework is valuable because pediatric success cannot be reduced to cosmetic height: visual development, head posture, amblyopia treatment, cooperation with examination, and the durability of symmetry must be assessed over time.

For children, the posterior approach is most defensible when the phenotype is favorable, the required elevation is limited, and the ocular surface and protective mechanisms are sound. General anesthesia removes intraoperative patient feedback, making a reproducible preoperative plan especially important. Families should understand that future growth or progression may require additional surgery and that long-term comparative evidence remains limited.

### 8.3 Neurogenic and myogenic ptosis

Neurogenic and myogenic ptosis traditionally directs attention away from MMCR because the underlying disorder may fluctuate or progress and because corneal protection may already be impaired. Nevertheless, a carefully selected stable patient may benefit from limited posterior elevation when levator function, Bell phenomenon, orbicularis strength, and surface reserve are adequate. The desired height is often deliberately conservative: clearing the visual axis without creating exposure is more important than maximizing MRD1.

The authors' recent series described MMCR with tarsectomy in chronic progressive external ophthalmoplegia (10). Its significance lies less in extending a routine indication than in demonstrating an individualized risk–benefit approach for progressive myogenic disease. Such patients require counseling that recurrence can reflect disease progression rather than failure of the initial operation. Staged or asymmetric correction may be appropriate, and postoperative lubrication and closure surveillance are central to the plan.

Fluctuating myasthenic ptosis, active third-nerve palsy, poor corneal sensation, weak orbicularis, and absent Bell phenomenon are warning features. Medical or neurological stabilization precedes surgery, and photographic measurements should be repeated at different times when variability is suspected. The decision to operate is strongest when the functional deficit is stable and reproducible and when the planned elevation preserves a generous safety margin.

### 8.4 Corneal grafts and other high-risk ocular surfaces

A corneal graft changes the balance of benefit and harm. Ptosis may restrict vision through an otherwise clear graft, yet posterior sutures, epithelial trauma, exposure, and infection can threaten the graft. Mangan and colleagues reported seven corneal-graft patients treated with MMCR with or without tarsectomy and a bandage contact lens placed at the end of surgery (50). The series supports feasibility when the graft is stable and protection is built into the procedure, but its size does not justify routine posterior surgery in every grafted eye.

Preoperative planning should be coordinated with the corneal surgeon when graft clarity, endothelial reserve, topical immunosuppression, or epithelial healing is uncertain. A low-risk surface in a reliable patient may tolerate a conventional MMCR with enhanced protection; a neurotrophic, repeatedly grafted, inflamed, or poorly closing eye may warrant a more conservative elevation, conjunctiva-sparing method, ELA, or no surgery. The visual benefit of raising the eyelid must be weighed against the possibility that exposure will reduce the very corneal clarity the operation is intended to reveal.

### 8.5 Combined and single-stage oculoplastic surgery

MMCR can be integrated with upper blepharoplasty, brow surgery, and selected ocular procedures, but combined surgery changes eyelid mechanics and surface risk. Skin excision should be conservative until the effect of posterior elevation on closure and brow position is understood. In patients with marked frontalis compensation, successful ptosis repair may lower the brow and alter the apparent amount of dermatochalasis; preoperative photographs with manual eyelid elevation can help demonstrate this interaction.

A single-stage approach may also reduce repeated anesthesia and recovery. In eight adults with ptosis and horizontal strabismus, Mangan and colleagues (51) performed strabismus surgery first, followed by MMCR with or without tarsectomy under general anesthesia. MMCR success was achieved in six of eight patients, strabismus success in all, and no revision operation was required; one patient had minimal transient lagophthalmos. The study is small and retrospective, but it shows how a predictable posterior plan can be useful when intraoperative eyelid adjustment is unavailable.

Combined surgery is most appropriate when each component has an independent indication and the sequence minimizes mechanical interference. Procedures that substantially alter vertical rectus position, eyelid retractors, orbital volume, or corneal exposure may be better staged. The patient should be counseled that efficiency is not the same as certainty: if the first procedure can materially change eyelid position, staging preserves diagnostic clarity.

### 8.6 A practical contemporary decision framework

The expanding literature does not support an indiscriminate expansion of MMCR. It supports a broader but more explicit selection process. Table 2 summarizes clinical settings in which a posterior approach may offer a practical advantage and the principal reason for caution.

**Table 2:** Contemporary clinical settings for MMCR and the safeguards that should accompany expanded indications.

Clinical context	Potential value of MMCR	Main limitation or safeguard
Typical aponeurotic ptosis	Short operation, preserved anterior lamella, reproducible preoperative planning	Confirm good levator function, closure, surface reserve, and realistic symmetry target
Residual or contralateral ptosis after ELA	Adds height without reopening a satisfactory anterior reconstruction	Use only when contour and crease are acceptable; larger height or contour defects favor ELA
Severe or congenital ptosis	Longer resection or selective tarsectomy may expand the achievable elevation	Selection depends on levator function and protection; evidence is mostly retrospective
Myogenic or neurogenic ptosis	Conservative visual-axis clearance without anterior dissection	Disease must be stable; prioritize Bell phenomenon, orbicularis strength, and exposure safety
Corneal graft or vulnerable surface	Can uncover a clear visual axis while preserving anterior anatomy	Coordinate corneal care, protect the graft, and avoid surgery when healing or closure reserve is poor
Combined surgery under general anesthesia	Preoperative algorithm avoids reliance on intraoperative patient cooperation	Stage procedures if the companion operation may materially change eyelid height or exposure

Figure 2 translates this framework into a simplified clinical decision pathway. MMCR is generally favored when levator function is preserved, posterior lamellar and ocular-surface reserve are adequate, and direct reconstruction of the levator aponeurosis, eyelid crease, or anterior lamella is not required. Phenylephrine testing informs the anticipated eyelid relationship but should not function as an absolute gatekeeper. Tarsectomy is reserved for selected eyelids requiring additional elevation when adequate tarsal height, eyelid closure, and ocular-surface reserve are present. Atypical etiologies, impaired protective mechanisms, or major structural abnormalities require a more conservative, staged, or alternative approach.

**Figure 2:**
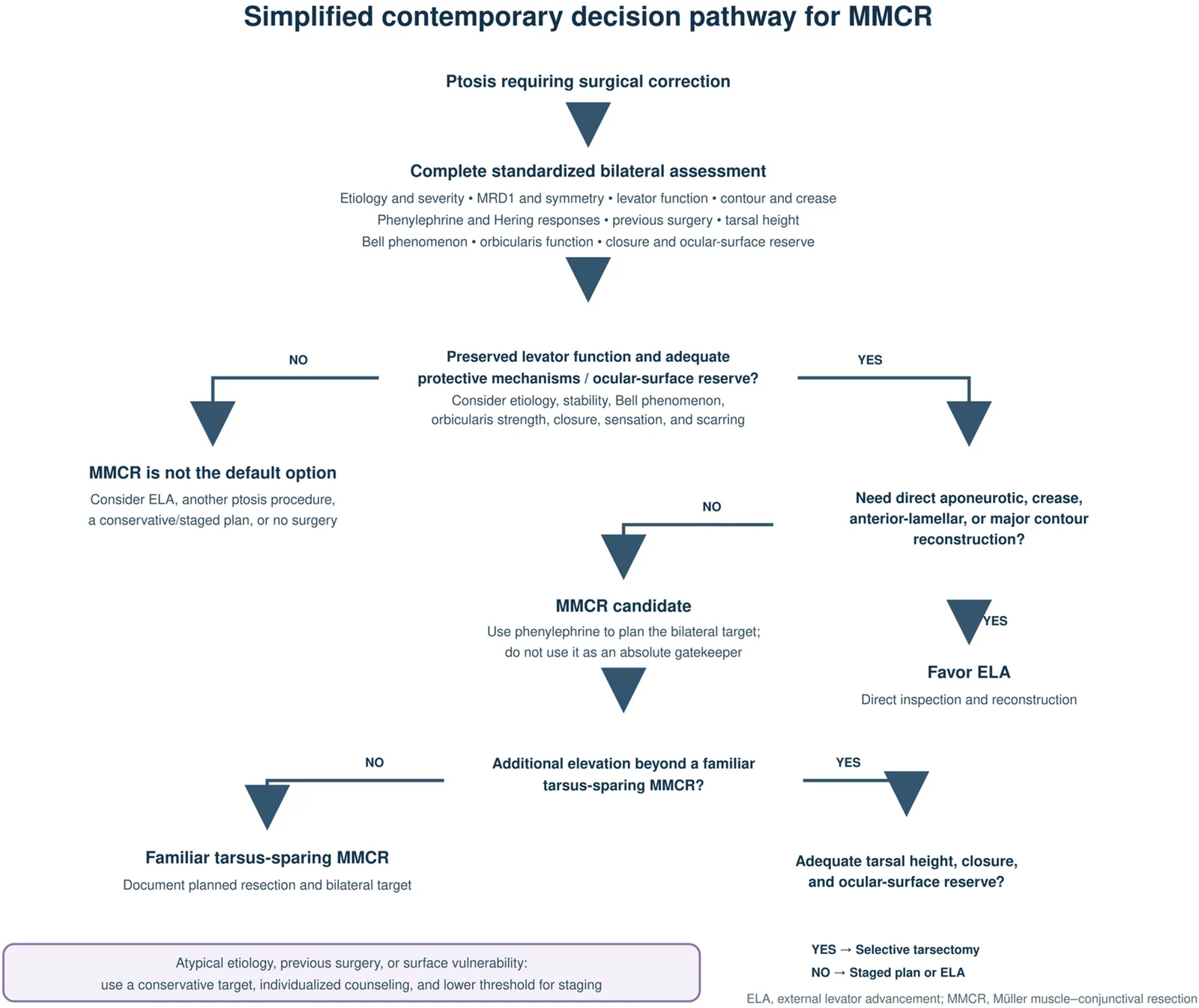
Simplified contemporary clinical decision pathway for MMCR. The pathway emphasizes baseline phenotype and protective mechanisms before phenylephrine-guided planning. It is intended as a clinical framework rather than a validated nomogram. ELA, external levator advancement; MMCR, Müller muscle–conjunctival resection.

## 9 Evidence gaps and priorities for future research

The modern MMCR literature is rich in technical innovation but limited by retrospective design, single-surgeon experience, small subgroups, inconsistent follow-up, and heterogeneous definitions of success. Even large comparisons are vulnerable to confounding because ELA is often selected for more severe or anatomically complex ptosis. Future comparative studies should therefore stratify or adjust for baseline MRD1, levator function, etiology, phenylephrine response, previous surgery, dermatochalasis, ethnicity, and ocular-surface risk.

We propose that future MMCR studies prespecify a primary positional endpoint assessed after eyelid position has stabilized, preferably no earlier than three months. Positional success should include a postoperative MRD1 within a prespecified target range, intereyelid asymmetry of 1 mm or less, and freedom from revision surgery. Each component should also be reported separately to prevent a composite endpoint from obscuring the reason for failure. Minimum secondary domains should include eyelid contour assessed using a predefined method, crease position, lagophthalmos and eyelid closure, corneal fluorescein staining, dry-eye symptoms, adverse events, and patient-reported functional and aesthetic benefit. Standardized bilateral photographs should be obtained in primary gaze with frontalis relaxation and consistent illumination and image scale. Early safety outcomes should be reported separately from stable effectiveness at three months or later and from longer-term durability. Results should be presented both per eyelid and per patient, with appropriate adjustment for within-patient correlation when both eyelids are included.

The phenylephrine test requires prospective clarification rather than a binary defense or rejection. Studies should record concentration, number of drops, timing, fixation eye, brow control, baseline variability, and the response of both eyelids. A multivariable model that integrates phenylephrine elevation with baseline MRD1, levator function, etiology, and previous surgery is more likely to be clinically useful than another isolated millimeter nomogram.

Technical studies should compare closed-clamp, open-sky, conjunctiva-sparing, sutureless, sealant-assisted, and tarsectomy-augmented procedures using the same endpoints. Imaging and histology could help determine which tissue components are actually advanced or shortened and whether those differences explain contour, dose response, and recurrence. Surgeon-level CUSUM monitoring may provide a practical method for training programs to distinguish procedural familiarity from stable clinical performance.

Finally, expanded indications need long-term, condition-specific data. Pediatric and congenital studies should include visual development and growth; myogenic series should separate surgical recurrence from disease progression; corneal-graft studies should report epithelial, graft, and endothelial outcomes; and revision studies should document the remaining conjunctiva and tarsus. These priorities would move the field from proof of technical feasibility toward evidence-based selection.

## 10 Conclusion

MMCR has evolved from a phenylephrine-dependent operation for mild aponeurotic ptosis into a versatile posterior platform. Its strengths are preservation of the anterior lamella, efficient and reproducible execution, favorable contour and revision outcomes in selected patients, and adaptability through tarsectomy, altered closure, conjunctiva-sparing dissection, and perioperative surface protection. Its weaknesses are equally clear: the mechanism and dose response are incompletely understood, phenylephrine is an imperfect predictor, and posterior tissue removal can be consequential in a vulnerable ocular surface.

The best contemporary use of MMCR is therefore neither formulaic nor unrestricted. Successful planning begins with the phenotype: levator function, contour, bilateral interaction, surface reserve, protective mechanisms, previous surgery, and the amount of elevation actually required. Phenylephrine contributes information but does not substitute for that assessment. Tarsectomy and repeat posterior surgery are selective tools, not automatic responses to a low measurement.

As oculoplastic surgery becomes more integrated, MMCR can serve as primary repair, a symmetry procedure after ELA, or one component of carefully planned combined care. The operation should ultimately be judged beyond eyelid height—by natural contour, stable bilateral symmetry, preserved corneal health, functional benefit, patient experience, and the avoidance of unnecessary revision. That broader definition of success is what allows posterior ptosis surgery to move beyond the eyelid while remaining safe at its surface.
